# Neuroanatomical and Neurocognitive Differences Between the Executive Functions in Child Sexual Offenders: A Systematic Review

**DOI:** 10.3390/brainsci15010038

**Published:** 2025-01-02

**Authors:** Yaiza Ara-García, Manuel Martí-Vilar, Laura Badenes-Ribera, Francisco González-Sala

**Affiliations:** 1 Department of Basic Psychology, Faculty of Psychology and Speech Therapy, Universitat de València, 46010 Valencia, Spain; agaryai@alumni.uv.es; 2 Department of Behavioral Science Methodology, Faculty of Psychology and Speech Therapy, Universitat de València, 46010 Valencia, Spain; laura.badenes@uv.es; 3 Department of Developmental and Educational Psychology, Faculty of Psychology and Speech Therapy, Universitat de València, 46010 Valencia, Spain; francisco.gonzalez-sala@uv.es

**Keywords:** pedophilia, child sexual abuse, neurocognition, neuroanatomy, executive functions

## Abstract

Background/Objectives: Studies on executive functions in child sex offenders relate their findings to the presence of pedophilia, but they are not able to distinguish between paraphilia and abuse. It is therefore this lack of a distinction that leads us to complement the existing information. Thus, the purpose of this review is to find all available evidence on the neurocognitive and neuroanatomical differences in executive functions among pedophilic and non-pedophilic child sex offenders, and non-offender pedophiles. Methods: The present review, in accordance with the PRISMA statement, ran a systematic search of three databases (Web of Science, Scopus and ProQuest). This search identified 5697 potential articles, but only 16 studies met all the inclusion criteria. Most of the studies were conducted in Europe, using a cross-sectional design with a convenience sample. Results: The results showed alterations in frontal, temporal and parietal structures related to executive functions (e.g., response inhibition) in child sexual offenders, regardless of the presence of pedophilia. Conclusions: In summary, there are differences in brain structure underlying executive functions related to child sexual abuse, but not to pedophilia as such.

## 1. Introduction

Sexual behavior is one of the most restricted and regulated biopsychosocial processes according to established social norms [1]. Therefore, any sexual affinity that does not correspond to genital or preparatory stimulation with phenotypically normal, physically mature and consenting human partners, within the cultural landscape, is found to be objectionable, abnormal or demeaning [2]. These atypical preferences correspond to what we understand as paraphilias.

One of the most common paraphilias is pedophilia, from the Greek “paidíon-” (children, prepubescent), “-philein/-philos” (love, inclination for, affinity) and “-ia” (quality of). It is translated as the quality of having sexual inclinations toward or an affinity for children or prepubescents. Pedophilia can be understood as an affective or romantic inclination to prepubertal individuals, as a psychological or neurological disorder, or as a sexual inclination including desires, fantasies, thoughts and behaviors [3,4].

On the other hand, child sexual abuse is a term that refers to people who maintain some kind of sexual approach to a minor without his or her consent, apparently related to the phenomenon of pedophilia. In fact, between 50% and 60% of child sex offenders are considered pedophiles [3,5], but this does not establish any causal relationship. Child sexual offenders usually have some characteristics in common, such as a difficulty relating to adults (affinity for children due to congruence with the emotional immaturity of minors), an inability to satisfy emotional and sexual needs, a need for power and control over the other, a lack of self-control (impulsivity), and behavioral disinhibition [6,7,8]. In addition to these, they may also present, in some cases, pedophilic inclinations.

How is child sexual abuse understood from the perspective of neurobiology and psychology?

So far, it has been suspected that pedophilia not acquired by brain injury or damage may be associated with neurodevelopmental disorders [9], although the origin of these alterations and their influence on pedophilia remain unclear. In contrast, numerous studies demonstrate alterations in child sex offenders at the behavioral level [3], at the personality level [10,11] and at the neurocognitive level. But it is not clear whether or not there is a distinction at the neurobiological level between pedophiles and child offenders, since a pedophilic inclination does not necessarily move a person to act according to his or her fantasies [12] and, even less so, to act without consent.

In studies related to child sexual abuse and pedophilia, different theories have been developed. Among the most popular ones related to executive functions is the “frontal lobe” theory [13,14,15,16,17]. This theory refers to alterations in the prefrontal, orbitofrontal and right and left dorsolateral cortex in male pedophiles, whether they have idiopathic (with unknown origin) or acquired (following brain damage) pedophilia [18]. However, these alterations have not allowed for discernment between pedophiles who have committed abuse and those who have not. This leads to the conclusion that the existing evidence does not clarify the role played by sexual preferences [19].

Another theory along the same lines, the motivation–facilitation model [5,20], explains the role that inhibitory control plays in the strong motivation that may appear in the presence of pedophilia. This control could be altered in individuals who go on to commit the act of sexually abusing a minor.

There is also a theory that combines the frontal lobe theory with the temporal lobe theory. The latter refers to the idea that dysfunctions in the temporal lobe could be behind hypersexuality, a frequent characteristic seen in sexual abusers [21]. Therefore, impulsive behavior could be explained by a dysfunction in frontal structures, and its relationship with the need to meet sexual demands could be explained by the simultaneous alteration of temporal structures. However, the possible influence of sexual preferences has not been clarified either. Along the same lines, it was precisely found that dysfunction in fronto-temporal regions was not associated with pedophilia, but with child sexual abuse exclusively [22,23]. Therefore, the relationship between brain dysfunctions associated with impulsive behavior and pedophilia remains a mystery.

Some authors have already become aware that most studies on pedophilia have included a sample of pedophiles who had sexually abused minors and, therefore, that the conclusions were associated with the paraphilia and not with the criminal act; i.e., they did not differentiate between pedophilia and sexual abuse [24,25,26]. One study conducted the first large-scale research effort that differentiated between pedophiles and non-pedophiles, as well as between individuals who had and had not committed sexual abuse. The study identified factors associated with different sexual practices (such as greater impulsivity in pedophile offenders) but did not specify the brain structures responsible for these factors. However, the study did suggest that executive functions may be altered in this type of behavior [24].

Executive functions are understood as all those higher cognitive structures and/or processes that direct, control and facilitate adaptation to new and complex situations, beyond habitual and automatic behaviors [27]. Some of these functions are working memory, behavioral self-control, response inhibition, processing speed and cognitive flexibility, among others [28].

The importance of this study lies in understanding the factors underlying child sexual abuse crimes, with the ultimate goal of helping professionals dedicated to the study of human behavior and its legal implications prevent and evaluate this type of behavior. The present study emphasizes the biological factor, determining whether there are neurobiological alterations in the executive functions of child sexual abusers and whether this phenomenon may be related to sexual preferences. For psychology professionals, it is necessary to detect all risk factors that may predispose individuals to a behavioral alteration of high impact on society, such as child sexual abuse, regardless of whether these factors are biological, psychological or social. A holistic understanding of the causes of child sexual abuse can increase prevention and direct intervention more efficiently in those individuals susceptible to committing this type of behavior.

Created following the PICO methodology, the research question on which the present study was based is as follows: are there differences in executive functions in people who have been convicted of pedophilia versus those who have not committed this type of crime? The aim of this systematic review was to find all the evidence in the literature of neurocognitive and neuroanatomical alterations related to executive functions in order to find differences between pedophile and non-pedophile samples, including both people who have sexually assaulted children and those who have not.

We structured and directed the review around our main question: will the findings differentiate pedophilic and non-pedophilic child sexual abusers?

## 2. Materials and Methods

We conducted a systematic review according to the Preferred Reporting Items for Systematic Reviews and Meta-analysis (PRISMA 2020) statement [29] (see Appendix A). This review was registered in PROSPERO (CRD42022380686), which confirmed that the present study was not duplicated and was free of any reporting bias.

### 2.1. Eligibility Criteria

Studies were included if they met the following criteria: (1) are empirical and original research; (2) are published in a peer-review scientific journal; (3) focus on altered sexual behavior toward minors (with and without consummated sexual abuse); (4) examine neurobiological and/or neurocognitive evidence in relation to executive functions; (5) use a sample of adults (i.e., over 18 years of age); (6) include a comparison between adults with different degrees of altered sexual behavior toward minors (taking into consideration pedophilia with and without consummated sexual abuse) and a control group of adults with no altered sexual behavior; (7) are written in English, Spanish, Catalan, French or German. These languages were included according to the authors’ domain. No geographical and time limits were established in the search for studies.

### 2.2. Search Strategy

Several search strategies were used. In order to establish a specific search strategy, we used the PICO reference [30]. We established inclusion criteria that defined the sample (non-probabilistic sampling by convenience that includes pedophilic and non-pedophilic men who had committed child sexual abuse or had not), the intervention or study objective (neurobiological and/or neurocognitive evidence in relation to executive functions) and the type of study to be compared (exploratory–comparative observational studies) and finally, compiled the results in order to draw conclusion

Then, an electronic search of all databases was performed between November 2022 and October 2024, obtaining representative results in the following three databases: Web of Science, Scopus and ProQuest. In this search, we used the following keywords in the English language: rape* OR rapist* OR “sexual abuse” OR “sexual offender” OR “sexual assault” OR pederast* OR pedophil* AND brain* OR neuroanatomic* OR neurocogni* OR neurobiologic* OR “executive function” OR “attentional control” OR “cognitive inhibition” OR “working memory” OR “inhibitory control” OR “cognitive flexibility. These keywords were combined in different ways and the search strategy was adjusted according to the specific requirements of each electronic database. A total of 5697 studies were identified across all databases (Web of Science: 2265 references; Scopus: 2025 references; and ProQuest: 1407 references).

Secondly, a manual review of reference lists of prior systematic reviews and meta-analyses [9,10,12,18,23,31,32] as well as a reference list of studies included in the present systematic review was performed to identify potential additional studies. This search strategy led us to identify 20 potential studies.

### 2.3. Study Selection Procedure

The selection procedure was carried out in two phases (pre-selection and selection). Each phase was carried out independently by one of two researchers. Disagreements were resolved through discussion, and, if necessary, a third researcher was consulted.

**Figure 1:**
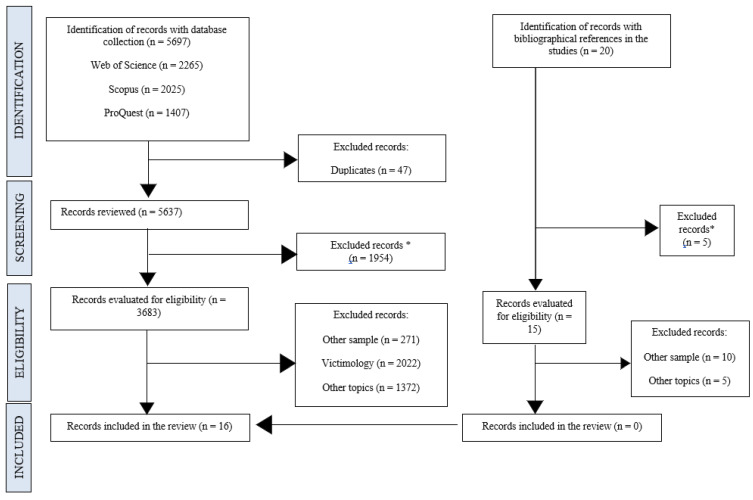
Prisma 2020 flow diagram of study selection process. * Records excluded because they do not follow the research format. They are written in other formats, mainly systematic reviews, meta-analyses and case studies.

Figure 1 displays a flowchart of the selection procedure. A total of 5697 potential articles were identified through electronic research and reviewed based on their titles and abstracts for eligibility. After 1954 references were removed because their abstracts did not match the inclusion criteria, 3683 full texts were examined. Of them, 3667 studies were excluded because they did not meet the inclusion criteria: 271 studies did not have a control sample, 2022 based their findings around victimology and 1372 studies only took some neurobiological and/or neurocognitive factors in executive functions but not as the main topic. Therefore, the search identified 16 published articles to be included for review, all written in English and published in peer-reviewed journals between 2009 and 2024.

In the manual literature search, a total of 20 potential articles were identified from the bibliographical references of the studies included in this review. The same eligibility criteria were applied, excluding 10 articles due to the sample type, 5 articles that did not include the topic of interest and another 5 that were reviews of the literature. Three of the studies were meta-analyses [9,18,32] and two were systematic reviews [12,31]. Finally, there were no articles left in the manual search to be included in the review.

### 2.4. Data Extraction

A protocol for data extraction from primary studies was established. Two independent researchers carried out data extraction in a systematic and standardized way. Once again, any disagreements were resolved by consensus, and a third researcher was consulted if required. The characteristics extracted were as follows: (1) authors and year of publication; (2) geographical location (country); (3) methodology (quantitative, qualitative or mixed); (4) research design (longitudinal vs. cross-sectional); (5) sampling method (randomized sample vs. convenience sample); (6) sample size; (7) setting (target population); (8) participants’ age; (9) gender distribution; (10) ethnicity distribution; (11) tools for assessing sexual offenders; (12) tools for assessing cool executive functions; and (13) type of pedophile (child abuse committed vs. child abuse not committed).

### 2.5. Risk of Bias and Quality Assessment

The quality of the primary studies was examined using the Grading of Recommendations, Assessment, Development and Evaluation, or GRADE [33,34]. The GRADE evaluates the quality of scientific evidence in the clinical and health sciences setting. The GRADE assesses six aspects through a series of items and conditions to be met: (1) the study design; (2) directness (patient population, diagnostic test, comparison of test); (3) the consistency of the results (similar in different studies); (4) the precision of the evidence (according to existing information in the literature); (5) the risk of bias (limitations in the study design and execution, the representativeness of the population that was intended to be sampled); and (6) quality level (all the studies show a moderate quality level). In this analysis, certainty is classified into four categories: high, moderate, low and very low. The high quality level shows high coincidence between the real effect and the estimated one; the moderate level may show a possibility of a gap between the estimated and the actual effect; the low quality level shows limited confidence in the estimate of the effect; and finally, the very low quality level shows very little coincidence between the actual effect and the estimated effect, with the actual effect being very different from the estimated effect. It should be added that randomized clinical trials are generally of high quality. In contrast, case studies, cohort studies and observational studies, among others, tend to have a low level of quality (see Appendix B).

## 3. Results

### 3.1. Characteristics of the Studies

The main characteristics of the studies included are displayed in Table 1. All studies were exploratory and comparative studies and used a non-probabilistic (convenience) sampling method. Most of the studies (10 out of 16 studies) were conducted in Europe [35,36,37,38,39,40,41,42,43,44]. Moreover, three were conducted entirely in the USA [45,46,47] and three were carried out jointly in the USA and Germany [48,49,50].

The studies’ sample sizes ranged from 29 participants [44] to 283 participants [49], with ages ranging approximately from 26.82 (SD = 9.28) to 44.86 years old (SD = 9.26). The participants were selected mainly from penitentiary centers (12 of the 16 items), social integration programs, outpatient or inpatient populations, online public forums, newspaper advertisements and e-mails. They were divided into pedophile men who had and had not committed child sexual abuse (or SO/NSO = sexual offender/non-sexual offender), non-pedophile men who had committed child sexual abuse and a control group of non-pedophile men who had not committed sexual abuse.

Most of the studies (14 of the 16 studies) analyzed cognitive constructs related to executive functions: inhibition or inhibitory control, planning, attention, or attentional control, working memory, processing fluency and speed, abstraction and abstract language, cognitive empathy, resistance to interference and cognitive flexibility. On the other hand, 2 out of the 16 studies analyzed neuroanatomical constructs related to executive functions, such as grey and white matter, brain surface area and laterality [48,49].

A wide variety of instruments were used to evaluate cognitive constructs. The most commonly used instruments were basic and functional magnetic resonance imaging (7 of the 16 studies), which is useful for creating images (static and moving) of the cervical tissue; the electroencephalogram [38], which obtains a pattern of the electrical activity of the brain; the Go/No-Go test (4 of 16 studies), which is mainly used to measure the capacity to maintain selective attention and inhibitory response control [51]; fractional anisotropy of white matter [49], a technique useful in diffusion tensor imaging (DTI) that quantifies the diffusion (or distribution of molecules across a concentration gradient) of water molecules in different brain tissues, specifically, in the white matter [52]; and finally, psychological tests or scales such as the WAIS-III (5 of 16 studies), among others, which measure cognitive abilities such as intelligence, attention and memory.

### 3.2. Neurocognitive and Neuroanatomical Alterations

Below are the main findings divided into two sections: neurocognitive or executive function alterations, and neuroanatomical alterations or brain structure changes (See Table 2).

#### 3.2.1. Neurocognitive Alterations

Evidence of this type of alteration was found specifically for attention control and interference in the pedophile population who had committed child sexual abuse. The findings showed a decrease in these capacities, due to abnormality and reduced brain activity in areas such as the superior parietal lobe and supramarginal gyrus [35,39,43,44].

Other relevant evidence is the inhibition of response or behavior in the pedophile population who had committed sexual abuse [35,36,37,38,39,40,41,44,45,48,49,50]. Both pedophiles and non-pedophiles who had committed sexual abuse of minors showed a lower inhibition of response and behavior. The brain areas with less activity that reinforced these findings are the left lower amygdala of the temporal cortex [36], the dorsal–medial prefrontal cortex [36,49], the left upper frontal cortex, the left caudal posterior cingulate cortex and the frontoparietal control network [37]. There was also a decrease in connectivity between amygdala–orbitofrontal cortex interaction [36] and the orbitofrontal cortex itself [40]. In contrast, hyperactivation in the left superior parietal lobe and precentral supramarginal gyrus [44] was seen in pedophiles who had committed sexual abuse.

On the other hand, pedophiles who had not committed sexual abuse of minors showed greater self-control and self-regulation [35,37], lower activation in the left and forehead region of the temporal upper gyrus and greater activation in the left nucleus accumbens [42] than pedophiles who had committed sexual abuse of minors.

The caudal and rostral part of the nucleus accumbens was also affected in the pedophile population that had committed sexual abuse [36,41,42], along with a visible reduction in gray matter. In contrast, in the left nucleus accumbens of pedophilic individuals who had not committed sexual abuse, an increase in activation could be observed [42].

In addition, pedophilic individuals who had committed sexual abuse showed greater cognitive flexibility (set-shifting) and perseverance [40,50] than non-pedophilic individuals who had committed sexual abuse. The latter also showed less capacity for the strategic use of working memory. In contrast, pedophilic individuals who had not committed sexual abuse exhibited less cognitive distortion, took fewer risks and were more prone to interference [39]. In spite of this, compared to the control group, pedophiles are characterized by having less awareness of error (related to risk assumption) [44].

Finally, learning deficiencies were found in pedophiles and non-pedophiles who had committed sexual abuse, as well as lower performance in processing speed (slowed visuomotor integration) in pedophiles (SO) and lower semantic performance in non-pedophiles (with SO) [46,47]. In non-pedophile sexual abusers, a greater display of impulsivity appeared [46].

#### 3.2.2. Neuroanatomical Alterations

Anatomically, a reduction in grey matter could be observed in pedophilic individuals who had committed child sexual abuse. The areas in which these reductions could be observed included the bilateral frontal lobe (including the premotor area, orbitofrontal cortex, frontal pole, inferior frontal junction, and dorsolateral prefrontal cortex), the parietal lobe (including the postcentral gyrus and medial precuneus), the temporal lobe (specifically in the inferior temporal gyrus, fusiform and parahippocampal gyrus) and the occipital lobe [41,43,48]. There was also a significant reduction in grey matter in the bilateral basal ganglia, bilateral cerebellum, bilateral medial cingulate and part of the hippocampus [48]. On the other hand, evidence was found of a reduction in white matter that appeared in the inferior surface area of the left prefrontal cortex and the right upper frontal cortex of pedophilic individuals who had committed child sexual abuse [49].

## 4. Discussion

The aim of the present systematic review was to find neurocognitive and neuroanatomical evidence underlying executive functions in pedophilic individuals including both those who have committed sexual abuse and those who have not, as well as in non-pedophilic individuals, again, including both those who have and those who have not committed sexual abuse. This study is relevant in that it can assist in determining the neurobiological alterations of executive functions in child sexual abusers and whether this phenomenon could be related to pedophilia. It should be added that the results should not be systematized, since the evaluation using the GRADE methodology shows evidence of a moderate degree of quality in the individual studies, so the results may represent an approach to an understanding of the phenomenon but not a strict conclusion.

Nevertheless, we discuss those approaches that we have been able to observe.

Until now, the scientific literature has shown that the phenomenon of pedophilia is a risk factor for sexual abuse. In fact, several studies have already hinted at causes associated with frontal and temporal neurocognitive impairments in pedophilic individuals [13,14,15,16,17,21]. What have not yet been understood rigorously are the differences in certain characteristics presented by these individuals; i.e., we do not know whether they present psychopathic traits or only paraphilia, or whether they have or have not committed sexual abuse against minors.

The studies have shown significant neurocognitive and neuroanatomical differences between sample groups, specifically between groups of perpetrators of child sexual abuse (see a summary in Table 3 and Table 4). The literature shows that pedophiles who commit child sexual abuse suffer alterations in attentional and interference control, an alteration that can be explained by a decrease in neuronal activity in the superior parietal lobe and the supramarginal gyrus. These areas are involved in the shift of attention through perceptual domains and in the reception of frontal information involved in this attentional shift [53]. This finding can be interpreted as a difficulty for pedophiles who commit sexual abuse in diverting their focal attention from the stimuli that represent their desire [43,44]. Other interpretations could include a difficulty in diverting attention from situations that predispose individuals to abuse or impulsivity (due to its relationship with the frontal lobe). Moreover, pedophiles who have committed sexual abuse show lower performance in processing speed [46,47]. This fact may be linked to a problem in neurodevelopment related to psychopathy.

One of the main findings regards response or behavioral inhibition in samples of individuals who have committed child sexual abuse, whether or not they were pedophilic individuals [35,36,37,38,39,40,41,44,45,48,49,50]. These findings include hypoactivation found in frontal brain structures [36,37,40,49]. We highlight orbitofrontal foreshortening and its interaction with the amygdala, since this decreased activity is reflected in difficulties in the processing and regulation of emotional content, including aspects such as morality and associations with stimulus reinforcement [36]. Moreover, the alteration in the amygdala corresponds to the findings of previous studies, in which considerable impairment in this structure was found in populations presenting with pedophilia [12,19].

The decrease can be interpreted as a risk factor for engaging in behaviors that require deeper aspects of morality and strong self-regulation of reinforcement, such as the sexual abuse or assault of minors. Moreover, it is related to reward processing and how rewards compensate for the value of punishment [54]. This fact may imply that sexually abusive behavior has a greater benefit than the cost of punishment for committing the action, which would result in biologically justified addictive behavior. It is also worth highlighting superior frontal foreshortening, which, in pedophiles who have committed sexual abuse, shows an important decrease, reflected in a limitation in working memory (essential for response inhibition) [37].

Furthermore, the alteration in the connection between the foreshortened temporal lobe and the left inferior amygdala is also reflected in a disruption of the functional integration of inhibitory regions. This finding could be another risk factor contributing to disinhibition in child sex offenders [36].

Other alterations observed include foreshortened parietal and temporal lobes [36,37,41,42,43,44,48,49]. Specifically, an atrophied or foreshortened parietal lobe [37] indicates a differentiation between offending and non-offending pedophiles. The non-offending pedophiles showed greater inhibitory control, which can be explained by taking into account the apparent normality of their frontal cortex and other structures involved, or by a greater capacity for elaborated self-control (bearing in mind that this sample was already undergoing therapeutic intervention). This evidence would support the motivation–facilitation theory [5,20], which determined that strong behavioral inhibition could control the strong motivation for sexually assaulting a victim. This group also showed greater self-referential processing [55]. In contrast, the offending pedophiles showed less inhibitory control due to decreased activity in parietal structures. Posterior cingulate foreshortening is related to conscientiousness, episodic memory, self-reference, and reactive deactivation when attention is directed outward [37]. Behavioral disinhibition is associated with a more deliberate response style, which could be explained by a lack of awareness of the immoral nature of the act or a strong need to obtain reinforcement. This explanation does not apply to non-offending pedophiles, as this group is characterized by strong behavioral inhibition due to their awareness of the immoral nature and the taboo connotation of their paraphilia. It is also possible that non-offending pedophiles, who do not present frontal disturbances, have greater self-control due to their conscientiousness of social repercussions [45] or due to their attentional control, which could help them to avoid all stimuli and risk factors [35].

As a first conclusion, it can be said that behavioral inhibition is independent of pedophilia and dependent on criminal sexual behavior. This is understandable given the impulsive nature of these types of offenders, as stated in “frontal lobe theory” [13,14,15,16,17].

Another finding is related to the reduction in white and grey matter [41,48,49]. This finding shows that certain areas (basal ganglia and cerebellum) form tank circuits with cortical structures that influence social cognition processes. Offending pedophiles showed an alteration in this area, in contrast to non-offending pedophiles [48], and, moreover, showed higher error-related positivity. This evidence, in contrast to other studies [56], indicates that white and gray matter alterations are associated with the criminal act and not with pedophilia, as found in previous studies [57]. Offending pedophiles also exhibited a reduction in grey matter in the prefrontal cortex, the nucleus accumbens and the temporal lobe that could explain the lack of affective empathic skills and inhibition of sex-related behavior [41], as stated in the “temporal lobe theory” [21]. Thus, there is further evidence to support the possibility that the non-offending pedophile sample’s ability of self-control allows them to have a greater awareness of the consequences of their actions.

Some studies have shown peculiarities in both the pedophilic and non-pedophilic sexual offender populations, such as a deficit in verbal memory performance [40]. This impairment, related to diminished verbal ability, is associated with higher levels of abuse due to the direct relationship it maintains with an inability to verbally mediate interpersonal conflicts [58]. According to these data, it can be inferred that child sexual offenders, regardless of whether or not they are pedophiles, are characterized by a framework of aggressiveness. Moreover, there is greater abstract reasoning in offenders (with and without pedophilia), a fact that may indicate the need for problem solving and the planning of their behavior [45].

Other evidence indirectly related to executive functions has been found and should be discussed before we conclude. In the pedophilic sample who had committed sexual abuse, we found a decrease in activity in the nucleus accumbens [36,41,42] in contrast to the non-offending pedophilic population. This decrease is related to an evaluation of emotional response and the prominence of emotional and motivational information about oneself and others, and is also involved in the initiation of goal-directed behavior and conflict control (moral and emotional mediation). Therefore, it could be said that these altered functions may also be behind sexual abuse.

### 4.1. Limitations and Future Research

Firstly, there was a limitation in the incorporation of articles that, in our first review of the summaries, appeared to meet all the inclusion criteria. Upon being analyzed in greater detail, they were excluded for not presenting sufficient information or for presenting unclear information regarding relationships with executive functions. This resulted in a greater time cost and a final review with fewer studies included. Moreover, most of the studies included were carried out in Europe; therefore, the conclusions inferred may not be significant in other populations.

Secondly, the type of sampling used was non-probabilistic for convenience, a sample type that does not guarantee the generalizability of the results, as stated in the objectives of this study. In addition, there was no homogeneous sample included in the studies. The same number of pedophile and non-pedophile samples were not obtained, regardless of whether they had committed child sexual abuse or not. This limitation is due to the difficulty in finding pedophiles who are not residing in rehabilitation programs or in clinical or penitentiary institutions. It is difficult for individuals in these condition to openly reveal their inclinations and, furthermore, to agree to participatein studies on their condition. Moreover, it is difficult to find a sample that has committed child sexual abuse and yet has not been deprived of its liberty, residing instead either in prisons or in clinical and reintegration centers. This could be a risk factor for bias in the performance of the tests or in the results obtained, since executive functions can be altered in long periods of liberty deprivation [59]. Samples that have received psychological treatment can also generate a certain bias, in comparison with other individuals with the same condition who have not received prior treatment. Furthermore, the differences between offending and non-offending pedophiles are not clear, as they may be influenced by social disaffection. However, it is also true that the variety in the sample has been able to resolve this uncertainty by producing clear results between the different groups as well as greatly reducing the possible range of social disaffection.

Thirdly, there is difficulty in interpreting the findings due to the fact that the evidence of child sexual abuse included does not differentiate between the types and severity of sexual abuse. The different variables that can complement sexual abuse, such as the violence perpetrated, could help in inferring one psychological profile or another. These variables are not taken into account in this review. Furthermore, the neurocognitive alterations found in child sex offenders and adult sex offenders are not differentiated. This fact could also be discerned in the activation of brain areas underlying executive functions.

As a final limitation of our review, we will add the impossibility of complementing the research with a meta-analysis, due to the lack of homogeneity in the methods and instruments used in each of the individual studies. In addition, the application of the GRADE methodology for the assessment of the risk of bias in individual studies showed evidence of a moderate degree of quality, leaving a gap in the analysis of the internal validity of the methodologies used, which hinders the conclusion of the work and indicates the importance of the use of a more specific questionnaire in the analysis of individual studies for future systematic reviews.

Thus, a first proposal for future research is to compare executive functions between child sex offenders and adult sex offenders in order to rule out the conclusion that the victim’s choice of age is determined by some kind of frontal neurocognitive alteration. In addition to a focus on the alterations in activation, an emphasis on the characteristics and connectivity of neural networks is also recommended.

It is known that there are certain factors that affect predisposition to child sexual abuse, such as the vulnerability and choice of the victim, distinguishing between pedophilia and hebephilia [60], an interest in children and disinterest in adults [61], a lack of social skills of the offender that are necessary to maintain social relationships with adults [3], low self-regulation of inhibition, which would mean low facilitation [5,20], and the predisposition and opportunistic backdrop, such as childcare, although these factors cannot apparently be explained by frontal executive alteration. It would also be advisable to review offenders who share these predisposing factors and see if they differ according to the age of the victim chosen. It is possible to hypothesize that child sexual offenders will have a greater lack of social skills with adults than adult sexual offenders.

Finally, the next alternative would be to compare pedophile child offenders and non-pedophile child offenders more exhaustively in order to assess whether there are alterations in other executive processes, such as planning and modus operandi. Lastly, as previously stated, it is necessary to differentiate between different types of sexual abuse (including more variables that can differentiate between abuse carried out by a pedophile and abuse perpetrated by a non-pedophile).

### 4.2. Implications for Clinical Practice and Social Policy

The present review therefore aims to improve the search for all those risk factors that predispose an individual to commit sexual abuse against minors. It is important to consider all the possible perspectives of a maladaptive behavioral phenomenon. This means including not only the underlying psychological or psychosocial phenomena, but also all the neurobiological characteristics that may indicate a previous alteration or susceptibility to the behavior. The social and judicial considerations of what these results represent must be taken into account. Our study does not conclude that there are other altered neurocognitive functions in pedophilic individuals apart from alterations in executive functioning. It is only concluded that pedophilia, from the point of view of the neurophysiology that supports behavioral self-control, is not a risk factor in child sexual abuse. It can be concluded that pedophilia is a risk factor in the choice of victim, but only if there is a cognitive predisposition to needing a victim, or if there is an abnormal pattern of sexual arousal caused by alterations in functional connectivity [32]. If there are no new neuroanatomical and neurocognitive findings in the relevant brain areas, pedophilia should not pose any criminal risk, from an executive point of view, in the sample used.

## 5. Conclusions

In conclusion, we cannot answer the initial question with complete certainty, as to whether the findings can differentiate pedophilic and non-pedophilic child sexual offenders. The quality assessment of the primary studies included in this analysis, using the GRADE system, showed that all the studies were of moderate quality, with consistent findings in several exploratory and comparative studies, despite some limitations related to study design and sample representativeness. However, we found that non-offending pedophiles show greater self-control, a greater awareness of the consequences of their actions, greater social and emotional cognition and less risk-taking behavior than those pedophiles who have committed sexual abuse toward children. Offending pedophiles do not differ from offending non-pedophiles in most executive functions, which leads to the conclusion that the neurocognitive alterations present are associated with the criminal act and not with the paraphilia, as concluded in other reviews [10,23]. Therefore, the “frontal lobe” theory [13,14,15,16,17] can be reaffirmed, but only under the condition of samples having committed child sexual abuse.

Such a conclusion shows that the executive frontal structures that are responsible for behavioral control, an awareness or self-reference of one’s own actions, and the act of letting oneself be driven by an impulse or avoiding stimuli (in relation to attention), as well as other processes, are not the ones that can explain why an individual with pedophilia may commit child sexual abuse. This is due to the fact that the functioning or alteration of these structures does not differ significantly from those of other individuals without the presence of paraphilia.

Therefore, it is possible that future studies may take into consideration and possibly find more evidence of the role that pedophilia plays in these type of behaviors.

## Figures and Tables

**Table 1:** Characteristics of individual studies.

Authors and Year	Country	Sample Design and Type	Sample			Construct	Instrument
			Size	Groups	Origin		
[45]	EEUU	Exploratory–comparative Non-probabilistic sampling by convenience	89	Middle-aged men 34.50 (ST = 8.3) (PEDs + SOs; n = 30), middle-aged men 31.70 (ST = 7.5) (NPEDs + SOs; n = 30) and middle-aged men 31.00 (ST = 7.2) (NSOs; n = 29).	Residents in intermediate programs between imprisonment and reintegration, and online public forum.	EF: commutation, inhibition, abstraction, working memory, fluency, planning and simple attention.	DEFKS, WAIS-III, WMS-III, SILS.
[35]	Germany	Exploratory–comparative Non-probabilistic sampling by convenience	93	Middle-aged men 42.09 (ST = 10.92) (PEDs + SOs; n = 22), middle-aged men 39.18 (ST = 8.77) (PEDs + NSOs; n = 11), middle-aged men 26.82 (ST = 9.28) (CG; n = 52 non-sexual offenders/n = 8 ASOs).	Outpatients, inpatients and online public forum.	EF: attentional control and sexual interest.	SMI iView X RED, NRP Picture set.
[36]	Germany	Exploratory–comparative Non-probabilistic sampling by convenience	40	Middle-aged men 43.67 (ST = 7.08) (PEDs + SOs; n = 12), middle-aged men 28.07 (ST = 5.71) (PEDs + NSOs; n = 14) and middle-aged men 32.86 (ST = 9.89) (CG; n = 14).	Correctional services and online public forum.	RSFC in default mode network (EF).	fMRI.
[37]	Germany	Exploratory–comparative Non-probabilistic sampling by convenience	117	Middle-aged men 38.25 (ST = 8.54) (PEDs + SOs; n = 40), middle-aged men 37.00 (ST = 8.84) (PEDs + NSOs; n = 37) and middle-aged men 36.65 (ST = 10.13) (CG; n = 40).	Internet platform and penitentiary services.	Neurobiological and behavioral inhibitory control.	MRI, Go/No-Go.
[48]	Germany, EEUU	Exploratory–comparative Non-probabilistic sampling by convenience	63	Middle-aged men 44.86 (ST = 9.26) (PEDs + SOs; n = 22), middle-aged men 44.24 (ST = 9.90) (NPEDs + SOs; n = 21) and middle-aged men 36.30 (ST = 5.35) (CG; n = 20).	Database of the Kiehl laboratory in the Mind Research Network (MRN), thanks to penitentiary institutions.	Gray matter.	MRI.
[49]	Germany, EEUU	Exploratory–comparative Non-probabilistic sampling by convenience	283	Middle-aged men 39.80 (ST = 9.00) (PEDs + SOs; n = 73), middle-aged men 34.20 (ST = 9.40) (PEDs + NSOs; n = 77) and middle-aged men 33.60 (10.2) (CG; n = 133).	Clinical centers, penitentiary institutions, online forum, advertisements and mailing lists.	Structural morphology of the encephalon: cortical groove, surface area and white matter FA.	MRI.
[50]	Germany, EEUU	Exploratory–comparative Non-probabilistic sampling by convenience	158	Middle-aged men 38.04 (ST = 8.62) (PEDs + SOs; n = 45), middle-aged men 36.51 (ST = 9.46) (PEDs + NSOs; n = 45), middle-aged men 40.26 (ST = 12.71) (NPEDs + SOs; n = 19) and middle-aged men 36.43 (ST = 6.70) (CG; n = 49).	Penitentiary institutions, German prevention project “Dunkelfield” and Internet platforms.	EF: impulsivity, planning and working memory.	CANTAB, IED, SOC, SWM.
[38]	Swiss, Germany	Exploratory–comparative Non-probabilistic sampling by convenience	61	Middle-aged men 35.30 (ST = 10.9) (PEDs + SOs; n = 21), middle-aged men 37.80 (ST = 9.7) (PEDs + NSOs; n = 19) and middle-aged men 30.80 (ST = 10.2) (CG; n = 21).	Penitentiary institutions and public newspapers.	Response inhibition and attentional control.	EEG, ERPs, Go/No-Go.
[39]	Swiss, Germany	Exploratory–comparative Non-probabilistic sampling by convenience	62	Middle-aged men 35.50 (ST = 10.64) (PEDs + SOs; n = 21), middle-aged men 37.19 (ST = 9.79) (PEDs + NSOs but convicted by sexual material; n = 20) and middle-aged men 30.76 (ST = 10.15) (CG; n = 21).	Penitentiary institutions, psychiatric hospitals and public newspapers.	IQ_F,_ IQ_C,_ alertness, orientation, risk-taking, resistance to interference, and episodic and working memory.	LPS, MWT-B, ANT, CGT, Stroop, CVLT, IAT, SMP, CRT task.
[40]	Germany	Exploratory–comparative Non-probabilistic sampling by convenience	63	Middle-aged men 38.70 (ST = 8.9) (PEDs + SOs; n = 15), middle-aged men 44.20 (ST = 7.9) (NPEDs + SOs; n = 15), middle-aged men 37.40 (ST = 9.1) (FC = 16) and middle-aged men 37.70 (ST = 10.2) (CG; n = 17).	Five penitentiary institutions and public newspapers.	EF: attentional control, cognitive flexibility, working memory, behavioral response change and inhibition and fluency.	WSCT, TMT-A, TMT-B, WMS-R, CBT, Go/No-Go, Tower of London.
[41]	Germany	Exploratory–comparative Non-probabilistic sampling by convenience	219	Middle-aged men 40.10 (ST = 9.1) (PEDs + SOs; n = 58), middle-aged men 34.40 (ST = 9.2) (PEDs + NSOs = 60) and middle-aged men 33.80 (ST = 10.5) (CG; n = 101).	Correctional institutions, clinical institutions, online advertisements, forum posts and email lists.	GI, impulsivity, empathy, inhibition and laterality.	WAIS-IV, BIS-11, IRI, SIS/SES, EHI.
[42]	Germany	Exploratory–comparative Non-probabilistic sampling by convenience	54	Middle-aged men 41.00 (ST = 9.00) (PEDs + SOs; n = 15), middle-aged men 38.80 (ST = 10.5) (PEDs + NSOs = 15) and middle-aged men 36.80 (ST = 12.9) (CG; n = 24).	Five penitentiary and clinical institutions, online advertisements, forum posts and email lists.	Intelligence (matrix reasoning, block design), laterality and cognitive empathy.	WAIS-IV, PD, IRI, fMRI.
[46]	EEUU	Exploratory–comparative Non-probabilistic sampling by convenience	60	Middle-aged men 34.30 (ST = 7.04) (PEDs + SOs; n = 20), middle-aged men 30.85 (ST = 6.32) (NPEDs + SOs; n = 20) and middle-aged men 36.70 (ST = 8.88) (CG; n = 20).	Three penitentiary institutions.	EF: intelligence, behavioral dyscontrol, resistance to interference, visual and auditory memory, and processing and motor speed.	WAIS-III, PIAT, SCWT, RV, RFFT, BDS, WMS-III.
[47]	EEUU	Exploratory–comparative Non-probabilistic sampling by convenience	60	Middle-aged men 34.15 (ST = 7.60) (PEDs + SOs; n = 20), middle-aged men 31.90 (7.79) (NPEDs + SOs = 20) and middle-aged men 29.70 (ST = 6.0) (CG; n = 20).	Residents in intermediary programs between prison and reintegration.	EF: working memory, processing speed and intelligence.	WAIS-III, SILS, WMS-III, FT, SS, DSC, ITT.
[43]	Poland	Exploratory–comparative Non-probabilistic sampling by convenience	42	Middle-aged men 43.80 (ST = 8.46) (PEDs + SOs; n = 11), middle-aged men 36.56 (ST = 8.8) (PEDs + NSOs = 14) and middle-aged men 32.24 (ST = 7.85) (CG; n = 17).	Department of Sexology at Nowowiejski Hospital (Warsaw) and social networks.	Emotional interference in cognitive control.	Go/No-Go, fMRI.
[44]	United Kingdom, Germany	Exploratory–comparative Non-probabilistic sampling by convenience	29	Middle-aged men 43.55 (ST = 11.58) (PEDs + SOs; n = 11), middle-aged men 33.25 (ST = 10.79) (PEDs + NSOs n = 8) and middle-aged men 37.70 (ST = 13.12) (CG; n = 10).	Penitentiary institutions, web page of the study, online forum, flyers and advertisements in public institutions.	Inhibition of interference.	Stroop interference NSIBP, error processing, post error slowing, fMRI

Note: PEDs = pedophilic offenders; SOs = sexual offenders; NPEDs = non-pedophilic offenders; NSOs = non-sexual offenders; CG = control group; ASOs = adult sexual offenders; FC = forensic control; IOs = incest offenders; DT = typical deviation; EFs = executive functions; DEFKS = Delis–Kaplan Executive Function Scale; WAIS-III/IV = Wechsler Adult Intelligence Scale, Third Edition or Fourth Edition; WMS-III = Wechsler Memory Scale, Third Edition; WMS-R = Wechsler Memory Scale Revised; SILS = Shipley Institute of Living Scales; SMI iView X RED = SensoMotoric Instruments BmGH; NRP Picture set = Not-Real-People Picture set; RSFC = resting-state functional brain connectivity; fMRI= functional magnetic resonance imaging; MRI = magnetic resonance imaging; FA = white matter fractional anisotropy; CANTAB = Cambridge Neuropsychological Test Automated Battery; IED = Intra/Extradimensional Set-Shift Task; SOC = Stockings of Cambridge Task; SWM = Spatial Working Memory Task; EEG = electroencephalograph; ERPs = Event-Related Potentials; IQ_F_ = fluid intelligence; IQ_C_ = crystallized intelligence; LPS = Leistungsprüfsystem; MWT-B = Mehrfachwahl-Wortschatz Test Version B; ANT = Attention Network Task; CGT = Cambridge Gambling Task; CVLT = California Verbal Learning Test; IAT = Implicit-Association Test; SMP = Semantic Misattribution Procedure; CRT task = Cognitive Reflection Test; WCST = Wisconsin Card Sorting Test; TMT-A and B = Trail Making Test Version A and B; CBT = Corsi Block Tapping Test; GI = global intelligence, BIS-11 = Barratt Impulsivity Scale Version 11; IRI = Interpersonal Reactivity Index; SIS/SES = Sexual Inhibition/Sexual Exhibition Scales; EHI = Edinburg Handedness Inventory; 2D:4D ratio = second-to-fourth digit ratio; PD = Personal Distress; PIAT = Peabody Individual Achievement Test; SCWT = Stroop Color and Word Test; RV = Recognition Vocabulary; RFFT = Ruff Figural Fluency Test; BDS-EV = Behavioral Dyscontrol Scale—Electronic Version; FT = Finger Tapping; SS = Symbol Search; DSC = Digit Symbol Coding; ITT = Inspection Time Task; NSIBP = neural Stroop interference and behavioral performance.

**Table 2:** Main neurocognitive and neuroanatomical findings of the studies.

Authors and Year	Sample	Results	Limitations
[45]	n = 89 Residents in reintegration and control programs	Higher abstract reasoning is observed in PEDs + SOs and NPEDs + SOs than in NSOs. Lower inhibition is observed in PEDs + SOs and NPEDs + SOs. PEDs + SOs show higher planning and development. PEDs + SOs show two-thirds below average interference, while NPEDs show one-third.	All offenders come from residential treatment programs with an interest in reintegrating into society. There is an insufficient sample size for more sophisticated statistical procedures (structural equations to examine contributing factors). There are poorly paired groups to present different characteristics. There is a lack of sensitivity in tests of executive functions (may detect inadequately).
[35]	n = 93 Clinical and control patients	PEDs + SOs show lower attentional control and therefore little interference control (inhibitory functions). PEDs + NSOs show a higher ability of self-control and self-regulation. Sexual interest in minors is the same in the two samples with pedophilic inclination.	A limitation may be the intelligence of ambulatory pedophile individuals as opposed to that of forensic pedophile individuals, with a higher score in ambulatory individuals. This study did not observe significant differences, but influence is not ruled out. The control groups are heterogeneous.
[36]	n = 40 Residents in prison and control	In PEDs + SOs, the lowest decrease in RSFC appears in the left medial OFC, followed by PEDs + NSOs and controls. In PEDs + SOs, there is also a decrease in RSFC in the DMPFC and in the rostral and caudal NAc. In addition, decreased connectivity appears in amygdala–OFC interaction. There is also a decreased connectivity of the ITC with the left amygdala.	The differences between PEDs + SOs and NSOs may be due to the age differences between the groups (PEDs + SOs exceeded 15 years in mean age), and, therefore, there may be bias in the comparative samples and lack of control for variables associated with experience It may influence the source of participant recruitment (the impact of age, gender preferences and incarceration).
[37]	n = 117 Residents in prison and control	PEDs + SOs show less activation of the medial parietal cortex (left caudal PCC) and the left SFC, and decreased FPCN activity. PEDs + NSOs have a neural mechanism with a compensatory function. Higher inhibitory control is observed in PEDs + NSOs. No differences between groups in the prefrontal area are observed.	Imprisonment may limit executive functions in the sample.
[48]	n = 63 Residents in prison and control	In PEDs + SOs, without significant differences with NSOs, a reduction in gray matter appears in the bilateral frontal lobe (premotor area, OFC, frontal pole, inferior frontal junction, DM/DLPFC), the parietal lobe (postcentral gyrus, precuneus medial, inferior and superior parietal lobe), the temporal lobe (inferior temporal, fusiform and parahippocampal gyrus) and the occipital lobe, and also in the bilateral basal ganglia (ventromedial and dorsolateral putamen, ventral caudate, nucleus accumbens, globus pallidus, left dorsal caudate), in the bilateral cerebellum, in the bilateral medial cingulate and in the hippocampus. Less gray matter is observed in the right hemisphere. There is more evidence in SOs than in pedophilia.	Age can be a risk factor (PEDs + SOs greater than PEDs + NSOs). PEDs + NSOs may not be considered SOs due to having been incarcerated before committing the act. There is a limited sample size. The assignment of subjects to each group according to criminological basis and the erroneous assignment of pedophiles (according to the DSM-IV) to the NPED group are limitations. This study does not include PEDs + NSOs.
[49]	n = 283 Residents in clinic and in prison and control	PEDs + SOs show reductions in cortical SA, white matter FA and CT. A lower SA is observed in the left CPF and right superior frontal cortex. Lower inhibitory control is observed in PEDs + SOs. The left DLPFC is uncoupled in a frontoparietal network in PEDs + SOs, which may indicate disinhibition.	Cortical mediation is a secondary statistical model; it may not be reliable. The sample differs in age and is strongly related to structural neuroimaging phenotypes. There is a reduced sample in neuroimaging studies. No “dose effect” is observed where the PED + NSO group is intermediary between PEDs + SOs and the CG. Cognitive function is not analyzed, so the components cannot be specified.
[50]	n = 158 Residents in prison and control	Less impulse control is observed in PEDs + SOs and NPEDs + SOs. PEDs + SOs show higher cognitive flexibility and change of scene. NPEDs + SOs show a lower capacity for the strategic use of working memory. Increasing age is associated with reduced response inhibition abilities (but no differences from the CG).	Imprisonment can be a limitation in the analysis of cognitive and executive functioning. There are possible confounding effects between psychiatric comorbidities and educational level. There is a lack of use of instruments for the diagnosis of sexual preference. The cross-sectional design does not allow us to clarify the impact of age on impulsivity and the onset of criminal behavior.
[38]	n = 61 Residents in prison and control	PEDs + SOs have less response inhibition. There are no differences between PEDs + SOs and PEDs + NSOs in behavioral self-control. Error-related positivity (PE) is greatly reduced in PEDs + SOs.	It is unclear whether criminal history and cognitive functioning co-vary. The sample is small and homogeneous (they do not differ in different pedophile behaviors).
[39]	n = 62 Residents in clinic and in prison and control	Deficits in errors in working memory are observed in PEDs + SOs. There is an observed correlation between pedophilia and low IQ. PEDs + NSOs exhibit less cognitive distortion, take less risks and are less prone to interference than PEDs + SOs.	The sample size is reduced. The absence of neuropsychological differences between SOs and the CG may be due to the influence of psychological factor such as low levels of antisociality (also associated with reduced levels of spatial intelligence. This study does not include PEDs + NSOs.
[40]	n = 63 Residents in prison and control	A dysfunction in response inhibition is observed in PEDs + SOs and NPEDs + SOs (OFC). NPEDs + SOs show little impulsivity and more time in taking tests. PEDs + SOs show higher perseverance and reactive cognitive flexibility. There are no significant differences between the groups in spontaneous cognitive flexibility. NPEDs + SOs show deficits in verbal memory performance.	Non-significant results comparing two groups may be biased by a lack of rigor in the post-hoc analysis (Bonferroni). The sample size is reduced.
[41]	n = 219 Residents in clinic and in prison and control	PEDs + SOs show a decrease in gray matter volume in the right temporal lobe compared to NPEDs + SOs. PEDs + SOs show a lower gray matter volume in the dorsomedial PFC and ACC. There are no gray matter differences between the groups in the right amygdala.	The cross-sectional design does not allow us to see causal explanations or predictions in the groups. There is a potential for social desirability bias in the PED + NSO sample (although there are clear differences, indicating that bias is low).
[42]	n = 54 Residents in clinic and in prison and control	Altered cognitive empathy neural processing is observed in PEDs + NSOs. Higher activation in the left NAc is observed. Higher activation in the STG is observed in PEDs + NSOs than in PEDs + SOs.	There are no correlations between behavior and brain activation between groups. It may be important to target the scanner’s task in activation rather than sensitivity. There is a possibility of social desirability bias in PEDs + NSOs (denying their status as SOs). There is no association between Pcu deactivation and PD level. A total of 2.7% of the correlations are above 0.50. The sample size is reduced. The cross-sectional design avoids future inferences.
[46]	n = 60 Residents in prison and control	Both PEDs and NPEDs show deficits in learning difficulties. PEDs + SOs show lower performance in information processing speed. NPEDs + SOs show lower performance in semantic knowledge and greater impulsivity.	There are errors in the analysis of the profiles in various neurocognitive domains, in the analysis of the reliability of the measures and in the differences between PEDs and NPEDs. Significant differences are not guaranteed by standard scores. There is a small sample size and a lack of rigorous definition of the groups. The results on FEs may be biased by the presence of general delinquent behavior and a low schooling level.
[47]	n = 60 Resident in reintegration and control programs	PEDs + SOs exhibit slower visual perception and visuomotor integration (lower processing speed) due to abnormalities in the white matter of the fronto-occipital fascicle [21].	The findings do not include cognitive elements in conjunction with neuroimaging. Biomarkers cannot be isolated from pedophilia alone. The sample size is reduced. There are differences between the groups, but no further information on this is provided. There is a ceiling and floor effect on some tasks (scores contrary to expectations). This study only examines visuo-perceptual processing speed.
[43]	n = 42 Clinical patients and control	PEDs + SOs show behavioral and neural abnormality during emotional interference in cognitive control (higher interference). PEDs + SOs show a decrease in the activity of the right DLPFC, with higher activity in PEDs + NSOs and the CG. There are no differences in cognitive control between SOs and the CG.	The sample size is reduced. Effects may be caused by a lower reactivity to emotional stimuli. There is subjective image evaluation.
[44]	n = 29 Residents in prison and control	PEDs + SOs show higher Stroop interference and a greater response time than NSOs and the CG (lower processing speed) due to the activation of the angular gyrus, left angular gyrus and cerebellum. PEDs + SOs have less inhibitory control of interference than NSOs (hyperactivation in the left SPL and precentral gyrus/SMG). PEDs + NSOs show higher activity in the left IFG, posterior cingulate, precuneus and medial temporal gyrus than PEDs + SOs. PEDs differ from the CG in their lack of error awareness.	The sample size is reduced. There is difficulty in differentiating criminal and non-criminal pedophiles. The effects of fMRI analysis with a uncorrected height threshold <0.001 may give false positives.

Note: PED = pedophilic offenders; NPED = non-pedophilic offenders; SO= sexual offenders; NSO = non-sexual offenders; CG = control group; RSFC = resting-state functional brain connectivity; OFC = orbitofrontal cortex; PFC = prefrontal cortex; ACC = anterior cingulate cortex; ITC = inferior temporal cortex; PCC = posterior cingulated cortex; FPCN = frontoparietal control network; SFC = superior frontal cortex; DM/DLPFC = dorsomedial/dorsolateral prefrontal cortex; SA = lower surface area; FA = fractional anisotropy; CT = Cortical Thickness; NAc = nucleus accumbens; STG = superior temporal gyrus; PD = Personal Distress; SPL = superior parietal lobe, SMG = supramarginal gyrus.

**Table 3:** Main neurocognitive differences between groups.

Executive Functions	Pedophile		Non-Pedophile	
	SO	NSO	SO	NSO (CG)
Abstract Reasoning	High	Regular	High	Regular
Inhibition of Response (Control)	Low	High	Low	Regular
Attentional Control	Low	Regular	Low	Regular
Processing Speed	Low	Regular	Regular	Regular
Semantic Performance	Regular	Regular	Low	Regular
Self-Control/Self-Regulation	Low	High	Low	Regular
Social and Emotional Cognition	Low	High	Low	Regular
Cognitive Flexibility, Perseverance and Set-Shifting	High	Regular	Low	Regular
Working Memory	Low	Regular	Low	Regular
Cognitive Distortion	High	Low	Regular	Regular
Awareness of Error (Risk Assumption)	Low	High	Low	Regular

Note: SO = sexual offender; NSO = non-sexual offender; CG = control group.

**Table 4:** Main neuroanatomical differences between groups.

Neuroanatomical Structures	Pedophilic		Non-Pedophilic	
	SO	NSO	SO	NSO (CG)
Left lower amygdala of temporal cortex, dorsal–medial prefrontal cortex, left upper frontal cortex, left caudal posterior cingulate cortex and frontoparietal control network	Low activity	Regular	Low activity	Regular
Superior parietal lobe and supramarginal gyrus	High activity	Low activity	Regular	Regular
Connectivity in amygdala–orbitofrontal cortex interaction	Low connectivity	Regular	Low connectivity	Regular
Temporal gyrus	Regular	Low activity	Regular	Regular
Angular gyrus and cerebellum				
Nucleus accumbens	Low activity	High activity	Low activity	Regular
Gray matter (*)	Reduction	Regular	Regular	Regular
White matter (**)	Reduction	Regular	Regular	Regular

Note: SO = sexual offender; NSO = non-sexual offender; CG = control group. (*) (**) see the specific areas in the Section 3.

## Data Availability

The authors are willing to make the data privately available to researchers who request the data.

## Appendix A

**Table A1:** PRISMA Protocol Guidelines (2020).

Items	Item n. º	Item Location
Title. Identifies the publication as a systematic review	1	1
Abstract. Structured summary	2	2
Introduction. Justification	3	3–6
Introduction. Objectives	4	6
Methods. Eligibility criteria	5	7
Methods. Sources of information	6	7
Methods. Research strategy	7	8–9
Methods. Study selection process	8	8–9
Methods. Data extraction process	9	9–10/34
Methods. List of data	10 a*	10
Methods. List of data (variables)	10 b*	10
Methods. Bias ratio in individual studies	11	10
Methods. Measures of the effect	12	-
Methods. Results synthesis (selection criteria)	13 a*	10
Methods. Results synthesis (required method)	13 b*	10
Methods. Results synthesis (presentation method)	13 c*	9–10
Methods. Results synthesis (justification)	13 d*	9–10
Methods. Results synthesis (heterogeneity)	13 e*	10
Methods. Results synthesis (robustness)	13 f*	10
Methods. Assessment by publication	14	10
Methods. Assessment of the certainty of the evidence	15	10
Results. Selection of the studies (research)	16 a*	10–11
Results. Selection of the studies (inclusive)	16 b*	10–11
Results. Characteristics of the studies	17	36–39
Results. Individual studies bias ratio	18	-
Results. Results of individual studies	19	40–42
Results. Results of the synthesis (characteristics)	20 a*	12–14
Results. Results of the synthesis (presentation)	20 b*	12–14
Results. Results of the synthesis (heterogeneity)	20 c*	12–14
Results. Results of the synthesis (robustness)	20 d*	12–14
Results. Publication bias	21	-
Results. Certainty of the evidence	22	34
Discussion. Interpretation of results	23 a*	14–19
Discussion. Limitations of the evidence	23 b*	19
Discussion. Limitations of the review process	23	19–22
Discussion. Implications and future lines of action	23 d*	22
Other information. Registry and protocol (registry)	24 a*	-
Other information. Registry and protocol (access)	24 b*	-
Other information. Registry and protocol (amendment)	24 c*	-
Other information. Funding	25	23
Other information. Conflict of interest	26	23
Availability of data, codes and other materials	27	23

Note: Page et al., 2021 [29]. * Items a–f are subsections of each item.

## Appendix B

**Table A2:** Evidence analysis—GRADE system.

	Quality Level	Study Design	Directness	Consistence	Precision	Risk of Bias
[45]	Moderate	*	Mod.	High	Mod.	Mod.
[35]	Moderate	*	Mod.	High	Mod.	Mod.
[36]	Moderate	*	Mod.	High	Mod.	Mod.
[37]	Moderate	*	Mod.	High	Mod.	Mod.
[48]	Moderate	*	Mod.	High	Mod.	Mod.
[49]	Moderate	*	Mod.	High	Mod.	Mod.
[50]	Moderate	*	Mod.	High	Mod.	Mod.
[38]	Moderate	*	Mod.	High	Mod.	Mod.
[39]	Moderate	*	Mod.	High	Mod.	Mod.
[40]	Moderate	*	Mod.	High	Mod.	Mod.
[41]	Moderate	*	Mod.	High	Mod.	Mod.
[42]	Moderate	*	Mod.	High	Mod.	Mod.
[46]	Moderate	*	Mod.	High	Mod.	Mod.
[47]	Moderate	*	Mod.	High	Mod.	Mod.
[43]	Moderate	*	Mod.	High	Mod.	Mod.
[44]	Moderate	*	Mod.	High	Mod.	Mod.

Note: The studies have been analyzed according to methodology characteristics. Mod = Moderate; * all investigations were exploratory and comparative studies and used a non-probabilistic (convenience) sampling method.
